# Production of Biodiesel from Lipid of Phytoplankton *Chaetoceros calcitrans* through Ultrasonic Method

**DOI:** 10.1155/2014/231361

**Published:** 2014-02-09

**Authors:** Raymond Kwangdinata, Indah Raya, Muhammad Zakir

**Affiliations:** Chemistry Department, Faculty of Mathematics and Natural Sciences, Hasanuddin University, Makassar 90245, Indonesia

## Abstract

A research on production of biodiesel from lipid of phytoplankton *Chaetoceros calcitrans* through ultrasonic method has been done. In this research, we carried out a series of phytoplankton cultures to determine the optimum time of growth and biodiesel synthesis process from phytoplankton lipids. Process of biodiesel synthesis consists of two steps, that is, isolation of phytoplankton lipids and biodiesel synthesis from those lipids. Oil isolation process was carried out by ultrasonic extraction method using ethanol 96%, while biodiesel synthesis was carried out by transesterification reaction using methanol and KOH catalyst under sonication. Weight of biodiesel yield per biomass *Chaetoceros calcitrans* is 35.35%. Characterization of biodiesel was well carried out in terms of physical properties which are density and viscosity and chemical properties which are FFA content, saponification value, and iodine value. These values meet the American Society for Testing and Materials (ASTM D6751) standard levels, except for the viscosity value which was 1.14 g*·*cm^−3^.

## 1. Introduction

Some problems which are very critical for the development of the industrial world today have happened. One is the energy crisis that must be resolved and addressed. This is due to the fact that continuous exploitation is not responsible for the fossil fuels being nonrenewable energy. This will have an impact on the scarcity of fossil fuels, thereby increasing the price of fuel oil (BBM) world. Diversification of energy is one solution to solve the problem, but the problem of saving the environment should also be considered, because almost every sector of public life cannot be separated from the use of fuel, which in fact resulted in environmental pollution, especially air pollution caused by vehicle emissions [1].

This situation has made most of the countries in the world (one of them is Indonesia) seek sources of alternative fuel that can be developed from other basic materials that are renewable and environment friendly [1]. Therefore, to meet the level of oil consumption and encourage the development and utilization of renewable alternative energy, biofuels (biofuels) such as biodiesel are used [2].

Indonesia is an archipelago with two-thirds of the area being the sea and the longest coastline in the world, which is 80791.42 km, is rich in aquatic biological resources, and which are very abundant both in kind and quantity. One of Indonesia's natural potentials is microalgae or phytoplankton [3].

Research on microalgae as a raw material of biodiesel, especially marine phytoplankton, has been carried out. However, research on the culture of phytoplankton that produced fats for biodiesel used as base material is still less common, particularly marine phytoplankton *Chaetoceros calcitrans*.

Marine phytoplankton *Chaetoceros calcitrans* has a fairly high fat content which is 14.60% to 16.40% by biomass dry weight, and phytoplankton species can reach the fat content of 39.80% of the dry weight in certain conditions (stress) [4].

For biodiesel production, lipids and fatty acids of natural sources have to be extracted from dry biomass of them like microalga biomass. Extraction methods such as ultrasound and microwave assisted were also used for oil extraction from natural sources. Biodiesel is a mixture of fatty acid alkyl esters obtained by transesterification (ester exchange reaction) of vegetable oils or animal fats. Transesterification is a multiple-step reaction, including three reversible steps in a series, where triglycerides are converted to diglycerides; then diglycerides are converted to monoglycerides, and monoglycerides are then converted to esters (biodiesel) and glycerol (by-product) [5].

The main problem in the biodiesel production process is that alcohol and oil as the main raw materials are not intermingled (immiscible). Stirring is a technique commonly used so that alcohol and oils can be mixed with each other so that the reaction can be run up to the formation of biodiesel, but mixing requires a relatively large energy [6].

From several studies that have been conducted, the use of ultrasonic waves has proven to accelerate the reaction, reducing the amount of catalyst used and reducing the ratio of oil to alcohol use than the reaction without the help of ultrasonic waves. This is due to the fact that ultrasonic wave energy arises from acoustic cavitation process (acoustic cavitation) which consists of the formation, growth, and collapse (implosive collapse) of the bubble formed. Ultrasonic waves cause the mechanical effects on the reaction to enlarge the surface area through microgap formation on the surface, accelerating dissolution, or increase the rate of mass transfer [7–9].

## 2. Materials and Methods

### 2.1. Materials

The materials used in this research work include phytoplankton cultures derived from *Chaetoceros calcitrans,* Bioinorganic Chemistry Laboratory, Hasanuddin University, ocean water from coastal areas, Makassar, sterilized, distilled water, Conway medium, sodium borax, KIO_3_, H_2_SO_4_, potassium iodide, methanol pa, potassium hydroxide, HCl, Na_2_S_2_O_3_·5H_2_O, anhydrous Na_2_SO_4_, oxalic acid, phenolphthalein indicator, indicator methyl orange, 96% ethanol, iodine (I_2_), starch, filter paper, label paper, and aluminum foil.

### 2.2. Apparatus

The apparatus used in this research work included glass tools which are generally used in the laboratory, jars made of cover glass, aerator, salinometer, centrifuge, haemocytometer, Japan Nikon microscopes SE model type 102, Olympus microscope SZX16, desiccators, pumps vacuum, Buchner funnel, water bath, water bath, Butchi rotary evaporator, blower, Oswald viscometer, burette 50 mL Pyrex, analytical balance, and ultrasonic equipment S 40 H Elmasonic.

### 2.3. Work Procedures

#### 2.3.1. Culture of Phytoplankton

Seawater is collected in a container and then sterilized subsequently measured by using a salinometer salinity and filtered using filter paper. Conway media added into sterile seawater and conditioned with aeration process CO_2_ gas then phytoplankton added into of those. After that, density of phytoplankton are calculated.

#### 2.3.2. Determinate Time of Phytoplankton Growth

Determination of phytoplankton growth pattern is done counting the number of cells per milliliter of medium every 24 hours. Samples are taken with a sterile pipette, dropped about 0.1–0.5 mL on haemocytometer, and then observed through a microscope [10].

#### 2.3.3. Isolation of Phytoplankton Lipid

Marine phytoplankton *Chaetoceros calcitrans* was dried in the oven, placed in erlenmeyer, added with 96% ethanol with a ratio of 1 : 6 w/v, and then extracted by means of an ultrasonic cleaner that operated at a frequency of 40 kHz. Ethanol extract was containing lipids that were separated by using a rotary evaporator.

#### 2.3.4. Synthesis Biodiesel through Ultrasonic Method

Pure lipids from marine phytoplankton *Chaetoceros calcitrans* already are obtained, inserted into the erlenmeyer, heated in an ultrasonic cleaner tool which is operated at a frequency of 40 kHz and a temperature of 50–60°C, and then mixed with a solution made of methanol (mole ratio of lipid : methanol = 1 : 12) and KOH catalyst (9 wt% oil) that has been stirred for 15 minutes. Time for the transesterification process was about 180 minutes. While the reaction takes place, the heating temperature should be maintained. Furthermore, the results of the transesterification were left for 3-4 days to form two phases. Then it separated, and followed by the addition of anhydrous Na_2_SO_4_ to the methyl ester to pull the rest of the water in the solution. The next stage was to separate Na_2_SO_4_ of biodiesel by using centrifuges. Supernatants in the form of methyl esters (biodiesel) were taken and then heated in an oven at a temperature of 70°C. Subsequently obtained pure biodiesel was then analyzed physical and chemical properties to determine the quality of the biodiesel.

#### 2.3.5. Analysis of Physical Properties

Analyses of the physical properties are density and viscosity. Density analysis procedures were carried out by the method ASTM D1475 and viscosity analyses were carried out by the method ASTM D445.

#### 2.3.6. Analysis of Chemical Properties

Analyses of the chemical properties are content of free fatty acid (% FFA), saponification value, and iodine value. Procedures of free fatty acids (% FFA) were based on the AOCS method Ca 5a-40, saponification value was based on AOCS method Cd 3-25, and value iodine was based on Wijs method.

## 3. Results and Discussions

### 3.1. The Growth Pattern of Marine Phytoplankton *Chaetoceros calcitrans*

**Figure 1:**
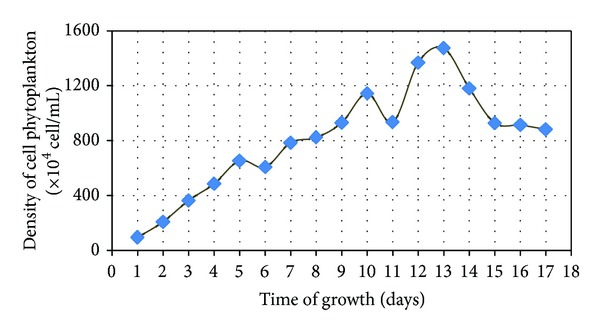
Chart patterns of marine phytoplankton growth *Chaetoceros calcitrans*.

Observations of marine phytoplankton growth pattern *Chaetoceros calcitrans* were done every 24 hours for 17 days by using the Conway medium as growth media in sterile seawater salinity adjusted and accompanied by the addition of vitamins to the media. The chart patterns of phytoplankton growth *Chaetoceros calcitrans* are shown in Figure 1.

Based on Figure 1, it could be seen that on day 1 till day 2 there was a phase of adaptation for phytoplankton *Chaetoceros calcitrans* to the growth medium. Later from day 3 to day 12 *Chaetoceros calcitrans* experienced a very rapid increase in population, known as the exponential phase. Furthermore at the stationary phase, the growth of rate began to slowdown, occurred on day 12 to day 13 which is unlike the previous days which occurred on day 3 to day 12. Then on day 13 until day 17 started a decline of phytoplankton populations *Chaetoceros calcitrans*. This phase is the phase where a decline in population mortality or decreased growth rate of phytoplankton occurs. Time optimal phytoplankton growth can be seen from the highest cell density of *Chaetoceros calcitrans*  1473.75 × 104 cells/mL which occurred on day 13.

### 3.2. Isolation Lipid of Phytoplankton *Chaetoceros calcitrans*

Early stages of biodiesel production from phytoplankton were isolation lipid of phytoplankton *Chaetoceros calcitrans* using ultrasonic extraction method. At this stage, solvent ethanol 96% is used. In this phase, the ultrasonic method plays an important role to destroy the cell wall composition of phytoplankton so that the function of ethanol will be more efficient in extracting lipids because it has the same polarity as the material to be extracted. Samples of dry biomass of phytoplankton *Chaetoceros calcitrans* at 25.89 grams and then extracted with 96% ethanol, extraction time 6 hours 50 minutes. The time is needed for extraction to belong a long time because the difficulty of cell wall damage.

Extracted in the form of lipids dissolved in 96% ethanol and then separated by means of solvent is evaporated until all ethanol 96% were used separately in order to obtain the pure lipid. Lipid weight of *Chaetoceros calcitrans* was 4.20 grams obtained so that the lipid content was 16.23% of biomass dry weight. Lipid content obtained from the phytoplankton species did not reach 50% of the dry biomass. This is due to the fact that not only does phytoplankton contain lipids, but there are also carbohydrates and protein.

### 3.3. Synthesis Biodiesel from Phytoplankton Lipid

**Figure 2:**
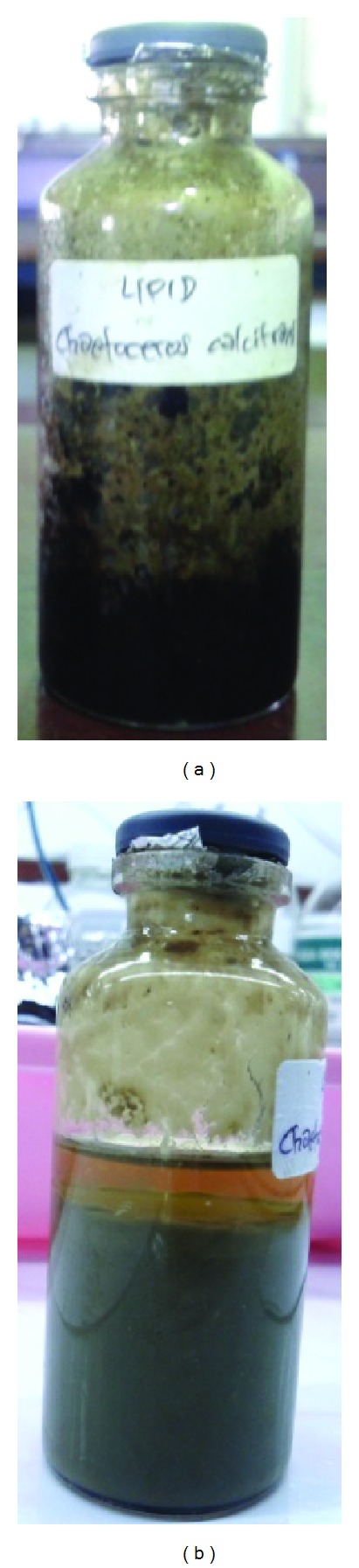
(a) Lipid of phytoplankton *Chaetoceros calcitrans*. (b) Result of transesterification reaction.

Synthesis biodiesel from phytoplankton lipid was done by transesterification using methanol (1 : 12). It was accelerated by the addition of KOH alkaline catalyst (9% of lipid weight). Time of transesterification reaction was around 180 minutes with a heating temperature of 50–60°C using an ultrasonic cleaner tool which is operated at a frequency of 40 kHz. Then, the reaction was left for 3-4 days to form two layers. The top layer was a layer of green biodiesel murky yellow, while the bottom layer is a layer of glycerol golden brown, which can be seen in Figure 2.

**Figure 3:**
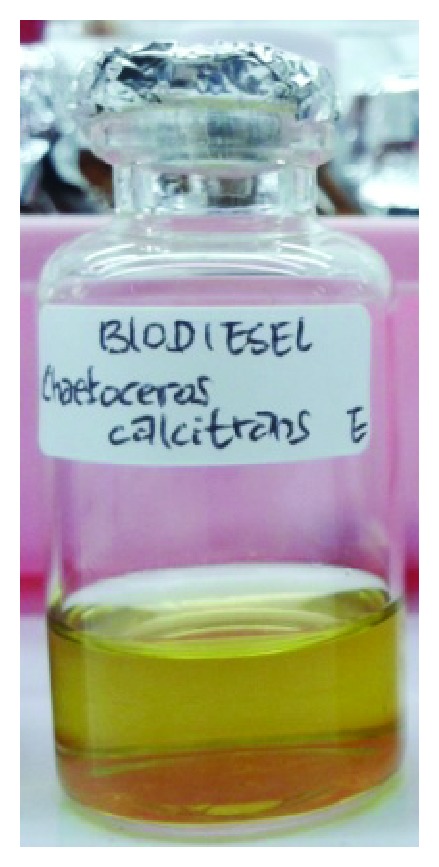
Biodiesel of phytoplankton *Chaetoceros calcitrans*.

Having obtained the two layers, the upper and lower layers were separated. The top layer was then centrifuged to remove impurities and glycerol which may end up at the time of separation. The remaining methanol in the biodiesel that does not react is removed by heating in an oven at a temperature of 70°C. Subsequently obtained pure biodiesel can be seen in Figure 3.

Weight of biodiesel is produced 9.15 grams with yield 35.35%. This is due to the fact that the fatty acids in the lipid component of phytoplankton have not reacted completely with methoxy ions in the transesterification reaction. Factors that could cause this are that the temperature and reaction time are not optimal. Biodiesel produced from phytoplankton also has a characteristic yellow color.

### 3.4. Analysis of Physical Properties

The next stage of the synthesis results of biodiesel from lipids phytoplankton *Chaetoceros calcitrans* through transesterification reaction was carried outly the characterization of physical properties based on the standard ASTM D6751. Test physical properties of biodiesel include analysis of density and viscosity. Density and viscosity analysis results can be seen in Table 1.

#### 3.4.1. Density Analysis

Biodiesel produced from lipid phytoplankton *Chaetoceros calcitrans* has a density value which was 0.88 g·cm^−3^ at a temperature of 40°C. The default value of 40°C density specified in ASTM D6751 is 0.82 to 0.90 g·cm^−3^. Density is one determinant of the quality of biodiesel as it pertains to the value of the generated heat and power diesel engines. The lower value of the density, the heating value, or combustion will also be higher [11].

When compared to the standard ASTM D6751, the biodiesel from the phytoplankton species can be said to be included in the range of density values that have been set.

#### 3.4.2. Viscosity Analysis

Viscosity is one of the standards in determining the quality of biodiesel and has a very important role in the process of fuel reinjection. Low viscosity value can lead to leakage of fuel injection pump and if too high can affect the work quickly and make carburetion injector fuel [11].

One of the causes of high and low viscosity grades is using the catalyst concentration and temperature. If concentration of catalyst is high, so the viscosity will decrease. This is because the concentration of excess catalyst will accelerate the breakdown of fatty esters triglyceride into three grades which will reduce the viscosity of 5–10%.

Kinematic viscosity results obtained in this research work were 1,14 cSt where the value which is smaller than the standard value of kinematic viscosity range recommended in ASTM D6751 is equal to 1.60 to 5.80 cSt. This is due to the persistence of residual methanol in the biodiesel that was contained in the viscosity value obtained which is rather small.

### 3.5. Analysis of Chemical Properties

Characterization of the chemical properties test was based on ASTM D6751 biodiesel made after the physical properties test is completed. Chemical properties of biodiesel test include the analysis of free fatty acid content (% FFA), saponification value, and iodine value. Results of analysis of free fatty acid (% FFA), saponification value, and iodine value can be seen in Table 2.

#### 3.5.1. Analysis of Free Fatty Acid Content (% FFA)

Free fatty acid value of biodiesel results of this research in which the value of 0.43% met the standard levels of free fatty acids/FFA (%) biodiesel recommended in ASTM D6751 is 0.45%.

Levels of free fatty acids that can cause deposition in combustion systems are also an indicator that the fuel can serve as a solvent which can lead to a reduction in the quality of the fuel system.

The higher the free fatty acids, the lower the quality of diesel fuel. High free fatty acids may also reduce the life of the pump and filter.

#### 3.5.2. Analysis of Saponification Value

Saponification number is defined as the milligrams of KOH required to neutralize one gram sample lipid or oil. The lower the molecular weight, the higher the saponification number and vice versa [12].

Saponification value results obtained of this research is 5.42 mg KOH/g where the value which is smaller than the standard value of saponification value in ASTM D6751 is less than 500 mg KOH/g. Based on these data biodiesel from *Chaetoceros calcitrans* phytoplankton species has a low saponification number and enters the biodiesel quality control set by ASTM D6751.

#### 3.5.3. Analysis of Iodine Value

Iodine numbers in biodiesel showed unsaturation level of the building blocks of biodiesel. On the one hand, the presence of unsaturated fatty compounds improved the performance of biodiesel at low temperatures because this compound has a melting point (melting point) that correlated with a lower cloud point and pour point was also low [13].

Biodiesel with high iodine numbers will produce esters with the flow and solidification at low temperature. Biodiesel which has a higher degree of unsaturation is not suitable for use as biodiesel because unsaturated molecules will react with oxygen from the atmosphere, be converted into peroxide crosslinking, result in the unsaturated, and cause biodiesel polymerized to form a similar plastic material, especially if the temperature increases. As a result, the diesel engine will not work properly and be damaged [14].

Biodiesel produced from lipid phytoplankton *Chaetoceros calcitrans* met the quality standards of ASTM D6751 iodine number is 20.90 g I_2_/100 g which met the quality standards iodine number in ASTM D6751 is less than 115 g I_2_/100 g.

## 4. Conclusion

Lipid phytoplankton *Chaetoceros calcitrans* can be isolated by ultrasonic extraction wherein the lipid content of *Chaetoceros calcitrans* is equal to 16.23% of biomass dry weight. Quantity of biodiesel synthesized from lipid phytoplankton *Chaetoceros calcitrans* through the ultrasonic method is equal to 9.15 grams with rendement 35.35%. Quality biodiesel from phytoplankton *Chaetoceros calcitrans* most had yet to meet the ASTM D6751 standard American Society for Testing and Materials (ASTM D6751). The parameter which is not met is the value of viscosity.

## Figures and Tables

**Table 1:** Result of density and viscosity analysis.

Density (g·cm^−3^)		Viscosity (cSt)	
Result of research	Standard ASTM D6751	Result of research	Standard ASTM D6751
0.88	0.82–0.90	1.14	1.60–5.80

**Table 2:** Results of analysis of free fatty acid (% FFA), saponification value, and iodine value.

Analysis	Result of research	Standard ASTM D6751
Free fatty acid content (% FFA)	0.43	<0.45
Saponification value (mg KOH/g)	5.42	<500
Iodine value (g I_2_/100 g)	20.90	<115
